# Bifunctional Metal Oleate as an Alternative Method to Remove Surface Oxide and Passivate Surface Defects of Aminophosphine-Based InP Quantum Dots

**DOI:** 10.3390/nano12030573

**Published:** 2022-02-08

**Authors:** Pin-Ru Chen, Minh-Son Hoang, Kuo-Yang Lai, Hsueh-Shih Chen

**Affiliations:** Department of Materials Science and Engineering, National Tsing Hua University, Hsinchu 30013, Taiwan; sophia801004@gmail.com (P.-R.C.); hmson.88@gmail.com (M.-S.H.); harry9703011345@gmail.com (K.-Y.L.)

**Keywords:** indium phosphide, oxidation, post-synthetic, quantum dots, passivation

## Abstract

The optical properties of indium phosphide (InP) quantum dots (QDs) are significantly influenced by their surface native oxides, which are generally removed by treating InP cores with hydrofluoric acid (HF). Besides the harmful health effects of HF, its etching may cause over-etching or QD size broadening, and surface oxidation can also reoccur rapidly. In the present study, a safer bifunctional metal oleate treatment was developed to simultaneously remove the surface oxide layer and passivate the surface defects for aminophosphine-based InP QDs. Compared to conventional HF etching, the bifunctional metal oleate was able to more efficiently remove the surface oxide of InP cores and effectively preserve the oxide-free surface, leading to a 20% narrower photoluminescence (PL) bandwidth after growing a ZnSe/ZnS shell. The metal oleate treatment is thus considered a greener and safer post-synthetic method to remove InP surface oxide and provide additional passivation to improve the optical properties of aminophosphine-based InP QDs, which could have potential in industrial mass production.

## 1. Introduction

As new-generation phosphor materials, colloidal quantum dots (QDs) have displayed attractive characteristics, such as size- and shape-dependent optical properties. However, the high toxicity of Cd and Pb seriously hinders the practical application of Cd- and Pb-based QDs [1,2]. Thus, there is a high demand for alternative materials. Indium phosphide (InP) QDs have been highlighted with low toxicity and optical characteristics competitive to existing Cd- or Pb-based QDs. Unfortunately, the synthesis of high-quality III-V InP QDs is still challenging and more complicated than II-VI QDs. In particular, as-synthesized InP QDs exhbit low photoluminescence quantum yields (PLQYs < 1%) and relatively broad emission bandwidths. Extensive efforts have been devoted to the fabrication of highly efficient InP QDs [3,4,5]; the overall properties of InP QDs still lag behind those of Cd-based QDs.

Incorporating QDs in applications such as light-emitting diodes [6,7,8,9,10,11], solar cells [12,13], bio labeling [14,15], and lasing [16,17] requires a thorough understanding of their behaviors in different environmental conditions. Surface chemistry plays a dominant role in nanoscaled QD behaviors because of a large surface-to-volume ratio [18,19]. The bare QD surface is usually faulty with surface defects states and dangling bonds. Therefore, a standard method to passivate the QD surface is to grow a wide-bandgap shell. However, even in a carefully controlled reaction excluding water and oxygen, surface oxidation could still affect the InP nucleation, growth, and shelling, leading to poor optical properties [20]. Hydrofluoric acid (HF) treatment has been used to remove the surface oxide layer and enhance the PLQY of QDs [21,22,23]. Unfortunately, the removed oxide layer of InP could regenerate rapidly when exposed to oxygen or moisture. In addition, HF hazards may hinder process practicability in mass production.

The HF etching method has been utilized to synthesize InP QDs for nearly two decades. Some milder alternative etchants such as NH_4_F or organic fluorides have been investigated, although HF treatment still has better results. In the present work, we developed a metal oleate method with bifunctionality. This process can simultaneously remove the surface oxide and passivate surface defects for InP QDs. It was occasionally found that a higher-concentration oleic acid in toluene could remove the surface oxides of aminophosphine-based InP QDs. We further introduced passivating metal ions to prepare a metal oleate with dual etching-passivation functions. With a metal oleate treatment, the PLQY of InP cores could be enhanced from ~0.1% to 9% before shell growth. Moreover, the oxide-free InP core surface could be preserved for a longer time, and the full-width at half maximum (FWHM) of PL peak of final core/shell InP/ZnSe/ZnS QDs could be reduced ~20%, compared with that of HF-treated ones. This metal oleate treatment is considered a greener, safer, and bifunctional route to remove surface oxides and improve the optical properties of InP QDs or cores at once, providing an alternative way for mass production of nanocrystals.

## 2. Materials and Methods

### 2.1. Materials

Indium chloride (InCl_3_, 99.999%), anhydrous zinc acetate (Zn(CH_3_COO)_2_, Zn(ac)_2_, 99.99%), cadmium oxide(CdO, 99.99%), sulfur powder (S, 99.98%), selenium powder (Se, 99.99%), oleic acid (OA, technical grade, 90%), 1-octadecene (ODE, technical grade, 90%), oleylamine (OAm, technical grade, 70%), trioctylphosphine (TOP, 97%), and hydrofluoric acid (HF, ACS reagent, 48%) were purchased from Sigma-Aldrich (St. Louis, MO, USA). Zinc chloride (ZnCl_2_, anhydrous, 99.95%) and tris(dimethylamino)phosphine ((DMA)_3_P, 97%) were purchased by Alfa Aesar (Ward Hill, MA, USA). All solvents were purchased from J. T. Baker (Radnor, PA, USA).

### 2.2. Synthesis of InP QDs

Synthesis of aminophosphine-based InP QDs was modified according to a previous work [24]. Briefly, 100 mg of InCl_3_ and 300 mg of ZnCl_2_ were mixed with 5 mL OAm in a three-neck flask inside a nitrogen-filled glovebox, then transferred to a Schelenk line. The mixture was degassed at 120 °C under vacuum conditions for 1 h, then heated to a reaction temperature under N_2_ atmosphere. At the reaction temperature of 200 °C, 0.45 mL (DMA)_3_P was quickly injected into the reaction mixture, and InP QD was allowed to grow for 20 min. At the end of the reaction, the reaction mixture was cooled to room temperature. Pristine InP QDs were those QDs that were directly collected from the reaction mixture without any postsynthetic treatment.

### 2.3. Metal Oleate Treatment

A zinc oleate (ZnOA) precursor was prepared by mixing 4 mmol of Zn(ac)_2_, 4 mL of OA, and 6 mL of ODE; and heating to an elevated temperature to form a clear solution. A cadmium oleate (CdOA) precursor was prepared by adding 4 mmol of CdO to replace Zn(ac)_2_; the rest of the procedures remained the same. According to the literature, pristine InP QDs were quantified by comparing the absorbance of quantitative aliquots at 413 nm with the intrinsic absorption coefficient of InP QDs at the same wavelength [24]. By measuring the absorbance of a known dilution of the reaction mixture in a short wavelength and using the intrinsic absorption coefficient of InP QDs, we can calculate the concentration of the InP QDs in the solution. The concentration of InP QDs in toluene solution was fixed at 10 mM for all experiments in this study. Metal oleate precursors were added in a corresponding ratio to the concentration of InP QDs and stirred for 3 min at room temperature. The resulting mixture was centrifuged by adding acetone to remove residual chemicals and the precipitated QDs were collected and redispersed in toluene for subsequent analyses.

### 2.4. HF Treatment

HF treatment was performed under atmospheric conditions, as reported in previous work [23]. The concentration of InP QDs in toluene solution was fixed at 10 mM. HF solution was prepared by adding a 10% H_2_O and 90% methanol to form an HF solution with a concentration of 0.5 M. The HF solution was added by an HF/InP ratio of 1000. The reaction mixture was stirred and illuminated by UV light with a reaction time of 10 min. The treated InP QDs were precipitated by centrifugation with acetone, and the precipitates were redispersed in toluene for further analyses. It should be noted that if the reaction time was prolonged over 10 min, the InP QDs would be gradually etched out, resulting in a transparent solution.

### 2.5. Growth of ZnSe/ZnS Shell

The InP cores were grown at 200 °C for 20 min in inert gas. The stock solutions were first prepared in a glovebox. Zn stock solution was prepared by dissolving 0.55 g Zn(ac)_2_ in 2 mL ODE and 2 mL OAm upon heating to form a transparent solution. Se stock solution was prepared by dissolving 2 mmol Se powder into 1 mL TOP. S stock solution was prepared by dissolving 4 mmol S powder into 2 mL TOP. Pristine InP QDs were first centrifuged with acetone to remove unreacted residuals in atmosphere. The precipitates were redispersed in 4 mL ODE and 1 mL OA. The Zn and Se stock solutions were injected into the pristine InP/ODE/OA mixture in inert gas. The mixture was then heated to 200 °C and reacted for 20 min. After that, the S stock solution was injected into the reaction mixture. Then, the mixture was heated to 220 °C and reacted for 20 min. Thus, pristine InP/ZnSe/ZnS QDs were prepared and collected from the reaction mixture for subsequent analyses in atmosphere. Shelled ZnOA-, CdOA-, and HF-InP QDs were prepared by replacing pristine InP QDs with ZnOA-, CdOA- and HF-InP QDs in the same concentration and same solvents, while the other experimental procedures remained the same.

### 2.6. Characterization

UV-vis absorption spectroscopy was measured by a UV-vis spectrometer (Hitachi U-3900, Tokyo, Japan) in 1 cm path length quartz cuvettes to acquire optical absorption spectra. PL spectra of InP QDs dispersed in toluene were recorded using a PL spectrometer (Horiba FluoroMax-4, Kyoto, Japan). PLQY of QDs was determined by comparing their fluorescence intensities with those of primary standard dye solution (Rhodamine 6G, QY = 95% in ethanol; Rhodamine 101, QY = 90% in ethanol) at the same optical density and excitation wavelength. The chemical states of each element in InP QDs were analyzed by X-ray photoelectron spectroscopy (XPS, PHI Quantera SXM). The binding energy was first calibrated from hydrocarbon contamination using the C 1s peak at 284.6 eV. XPS quantification was performed based on Scofield’s relative sensitivity factors (RSF). Fourier-transform infrared (FTIR) spectra were collected by Brucker Tensor 27 using the KBr pellet method. QD structure was studied via X-ray Diffraction (XRD) by X-ray diffractometer (Bruker D2 phaser) with Cu Kα radiation. XRD samples were prepared by drop-casting QD solution onto a glass slide. ^31^P solid-state nuclear magnetic resonance (SSNMR) spectroscopy was recorded by Bruker Avance III 400. To prevent further oxidation, all samples were packed into 4 mm rotors and sealed with an airtight rubber spacer inside the glovebox. Time-resolved PL (TRPL) spectra were obtained from PicoHarp300 with TTTR Mode and PHR 800 router (PicoQuant, Berlin, Germany).

## 3. Results and Discussion

### 3.1. Conventional HF Treatment to Remove Surface Oxide of InP QDs

InP QDs prepared from aminophosphines instead of tris(trimethylsilyl)phosphine ((TMS)_3_P) is a safer method, and can be feasibly prepared from InCl_3_, ZnCl_2_, and (DMA)_3_P in OAm. It should be noted that the addition of ZnCl_2_ helps reduce the size distribution of InP QDs, and the minor absorption of ZnCl_2_ on the QD surface would not form an InZnP alloy QDs [24]. According to elemental analysis from ICP-MS, the as-synthesized InP QDs have In/P/Zn atomic ratio of 50.1/45.5/4.4. Pristine InP QDs (i.e., as-synthesized InP QDs without any post-synthetic treatment) generally have a low PLQY (e.g., <1%) because of the surface dangling bonds or defect states that have been observed in III-V InP QDs [21,25,26].

**Figure 1:**
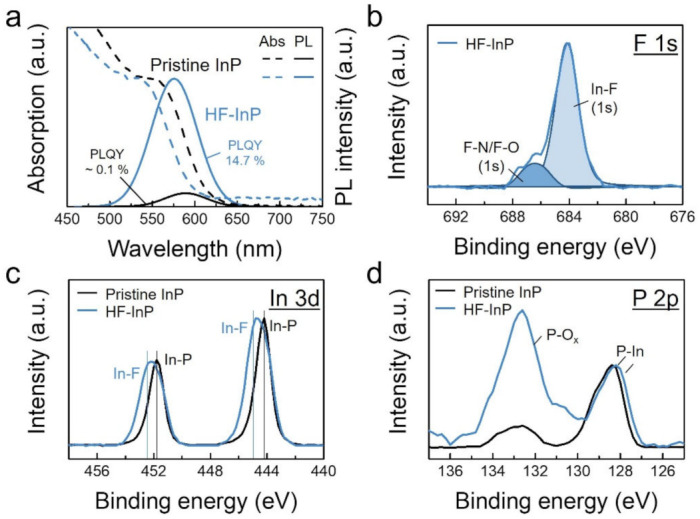
Comparison of pristine InP (black curve) and HF-treated (HF-) InP (blue curve) QDs. (**a**) Normalized PL and optical absorption spectra. The first excitonic absorption and PL peaks blueshift 14 nm and 18 nm, respectively, after the HF etching process. Corresponding XPS (**b**) F 1s, (**c**) In 3d and (**d**) P 2p spectra.

Post-synthetic HF treatment is commonly utilized to improve the optical properties of InP QDs via the removal of their surface oxides and defects [23]. As shown in Figure 1a, the PLQY of pristine InP and HF-treated InP (HF-InP) QDs are ~0.1% and 14.7%, respectively. Besides the PLQY improvement, the HF treatment also results in significant blueshifts (>10 nm) in both optical absorption and PL peaks because of size reduction in the etched InP cores. In addition, the absorption curve of HF-InP QDs slightly broadens, indicating that the HF etching causes a size reduction and extends size distribution. The PL FWHM also increases from 57 to 63 nm after the HF etching process.

The surface chemistry of HF-InP QDs is examined by XPS analysis. XPS survey spectra of pristine and HF-InP QDs are shown in Figure S1. Figure 1b shows the F 1s spectrum of the HF-InP QD sample. HF-InP QDs have a strong In-F intensity at 684.2 eV, implying HF treatment effectively passivates the InP surface by In-F binding. A small peak at 684.6 eV is attributed to organic fluorine such as F-N or F-O, which would be by-products generated when HF reacts with surface OAm ligands [27,28]. Figure 1c shows In 3d spectra of the pristine and HF-InP QDs. Compared with pristine InP, a relatively broad In 3d doublet of the HF-InP QD sample also suggests that HF-InP QDs are mainly passivated with In-F, as reported previously [29]. Figure 1d shows P 2p spectra of the samples, clearly demonstrating that the HF-InP QD sample has a stronger P-O_x_ peak at ~133 eV in addition to P-In binding at 128.5 eV [20]. There is no significant peak correlating to the P-F binding (e.g., ~137 eV) observed in the XPS survey spectra (Figure S1). [21]. It is thought that HF etches the surface oxides on InP, and P-H binding might form right after the HF treatment. No P-H binding can be identified in XPS because the formed P-H binding might not be stable enough, and thus P-O_x_ compounds form in the atmosphere during sample transportation to XPS measurement. Re-oxidation of HF-treated InP after exposure to the atmosphere has also been observed previously [21,30,31,32]. Oxidation of P-H binding to P-O_x_ may be supported by calculated Gibbs free energy of oxidation of phosphine that is highly negative, as listed in Tables S1–S3.

The HF treatment can lift the PLQY of HF-InP QDs (~150 folds), along with a rapid PLQY decay of HF-InP QDs in a couple of hours, showing that the HF passivation only has a temporary effect but cannot be sustained for a long time. Furthermore, the HF-InP QDs have a higher P-O_x_/P-In intensity ratio than those of pristine InP QDs, revealing that the oxidation of HF-InP QDs in the atmosphere mainly occurs at the P sites, while In-F passivation could still exist. A similar result is observed in silicon with SiO_x_ native oxides, which can be effectively removed by HF and form Si-H passivation at the Si surface, but the Si-H passivation also cannot be sustained for a long time (e.g., a couple of minutes) [33].

### 3.2. Alternative Method to Remove Surface Oxide by Bifunctional Metal Oleate Treatment

**Figure 2:**
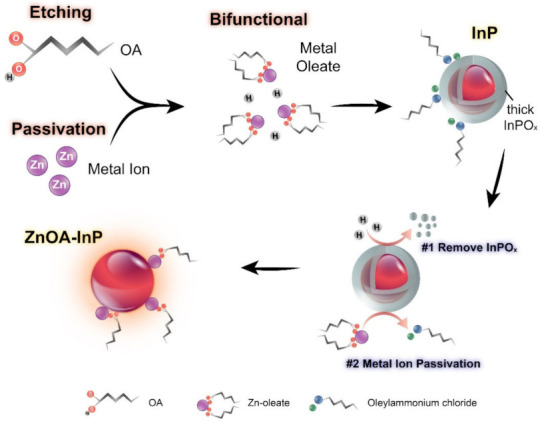
A sketch illustrating the bifunctional metal oleate to both passivate InP surface and etch surface oxide layer.

Although HF treatment can effectively remove the oxides from InP QDs, rapid re-oxidation and HF hazards may limit processability and practicability in mass production. In the present study, we found that OA is able to etch aminophosphine-based InP QDs when the QDs are treated with high-concentration OA in toluene solution for a certain amount of time, as shown by a broadened and blue-shifted absorption spectrum in Figure S2. The OA etching appears to be weaker and slower than HF etching, and the resulting PLQY is not obviously enhanced by the OA etching method. Thus, some passivation metal ions are introduced into OA. The mixture is a bifunctional reagent that can simultaneously remove the surface oxide layer and passivate InP QD surface, as illustrated in Figure 2. It is known that OA can provide protons to dissolve the surface oxides, while both metal ions and oleate passivate the InP surface. Comparison between control samples after post-treatment with only metal salts and with only OA are discussed in Figure S2.

**Figure 3:**
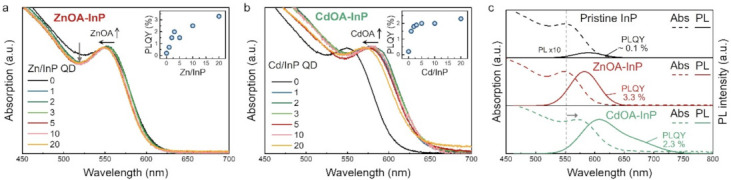
Comparison of optical absorption and PL spectra for treated InP QDs in different metal ions/QD concentrations. (**a**) ZnOA treated (ZnOA-) and (**b**) CdOA treated (CdOA-) InP QDs with various metal oleate concentration. Insets of (**a**,**b**) are PLQYs of corresponding ZnOA- and CdOA-InP QDs. (**c**) PL and optical absorption spectra of pristine, ZnOA- and CdOA-InP QDs in a metal oleate to InP ratio of 20.

Both zinc oleate precursor (ZnOA) and cadmium oleate precursor (CdOA) are used in this study. Pristine InP QDs are treated with various concentrations of ZnOA and CdOA, respectively, with Zn/InP QD and Cd/InP QD ratios from 0 to 20, as shown in Figure 3a,b. When InP QDs are treated with a higher concentration of ZnOA or CdOA, the optical absorption of ZnOA-treated (ZnOA-) and CdOA-treated (CdOA-) InP QDs blueshifts indicate a reduction in InP QD size caused by the etching effect. The corresponding PL spectra also have a similar blueshift trend (Figure S3). InPO_x_ on InP QDs could be etched by either ZnOA or CdOA, as described in Equations (1) and (2) [34,35,36]:

InPO_4_ + 3H^+^ → In^3+^ + H_3_PO_4_ (1)

In(PO_3_)_3_ + 3H_2_O + 3H^+^ → In^3+^ + 3H_3_PO_4_ (2)

The first excitonic absorption peak of InP QDs becomes narrower after the ZnOA treatment, suggesting a narrower size distribution (Figure 3a). The PLQY of ZnOA-InP QDs increases up to 3.3% (enhanced ~33 folds) with the increase in ZnOA concentration, as shown by the inset in Figure 3a. Enhanced PLQY is also observed for CdOA-InP QDs (up to 2.3%, ~ 23 folds), although their size distribution has uncertain change (Figure 3b). Increased PLQY infers that the metal oleates passivate the QD surface.

Figure 3c shows the comparison of optical absorption and PL spectra of pristine InP QDs, ZnOA-InP, and CdOA-InP QDs in a Zn/InP QD and Cd/InP QD ratio of 20, respectively. Pristine InP QDs exhibit the first excitonic absorption at 549 nm, PL at 589 nm, and relatively low PLQY ~ 0.1%. On the other hand, ZnOA-InP QDs show the absorption peak at 547 nm (2 nm blueshift), PL peak at 582 nm (7 nm blueshift), and an improved PLQY of 3.3%. Moreover, CdOA-InP QDs demonstrate large spectral red-shifts in the absorption peak at 569 nm (~20 nm redshift) and PL peak at 607 nm (~18 nm redshift), together with a slight increase in the PLQY (~2.3%). Notably, CdOA-InP QDs exhibit a broad defect emission tailing toward lower energy, implying some defect states might be generated at the QD surface, leading to a lower PLQY than that of ZnOA-InP QDs [37,38,39]. Likewise, the large red-shifts in optical spectral peaks of CdOA-InP QDs are ascribed to the delocalization of excitons to the surface defect states [37]. The existence of crystalline defects could be supported by XRD analysis, as depicted in Figure S4, where CdOA-InP QDs possess a relatively weak diffraction intensity, inferring a lower crystallinity. Accordingly, the CdOA treatment might over-etch InP QDs and produce additional surface defects. A previous study reported that the reaction of Lewis acid (Zn^2+^ and Cd^2+^) with OA-capped InP QDs at an elevated temperature of 200 °C could improve their PLQY but other In_2_O_3_ are formed as by-products [40], which is a distinct mechanism compared with the current study. The surface oxide removal effect by metal oleates in this study may have originated from the different surface chemistry of aminophosphine-based InP QDs.

### 3.3. Surface Chemistry of ZnOA- and CdOA-InP QDs

**Figure 4:**
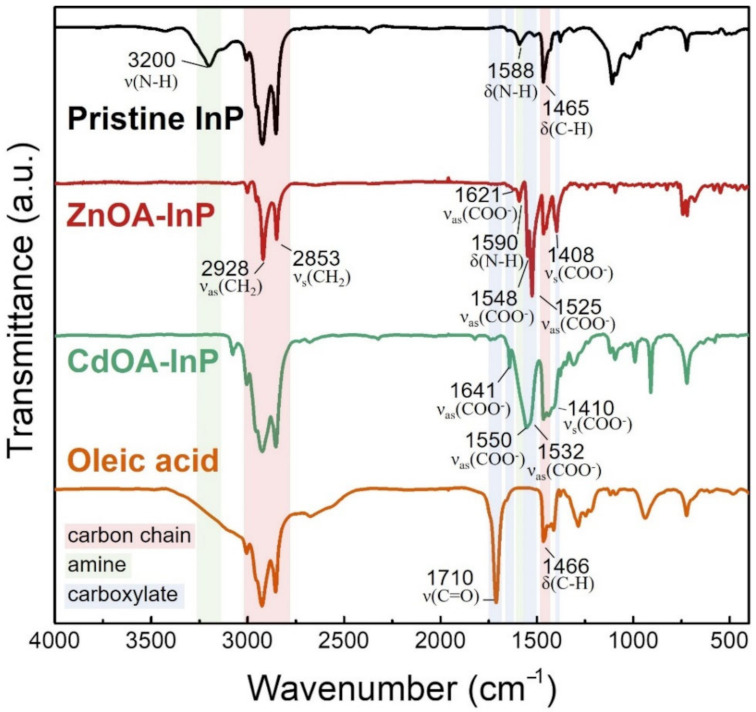
FTIR spectra of pristine, ZnOA- and CdOA-InP QDs, and OA to determine the surface ligands on QDs.

ZnOA and CdOA surface passivation on InP QDs is studied by FTIR, as shown in Figure 4, in which all InP samples show strong symmetric and asymmetric stretching vibration of CH_2_ group in the range of 2850 to 2950 cm^−1^, indicating a long alkyl ligand capping on the QD surface. The pristine InP QDs show a resonance peak at 3200 cm^−1^ originating from N-H stretching, proving that the QD surface is covered with amine-related species [41]. For ZnOA- and CdOA-InP QDs, the absence of the resonance peak at 3000–3500 cm^−1^ confirms the removal of ligands originally on the QDs. The existence of symmetric ((ν_s_(COO^−^) at 1408–1410 cm^−1^) and asymmetric stretching (ν_as_(COO^−^) at 1525–1641 cm^−1^) in carboxylate groups suggest that both ZnOA- and CdOA-InP QDs have OA surface ligands. Lack of a resonance peak at 1710 cm^−1^ relating to C=O stretching of free OA molecules indicates that the oleates should bind to the metal sites on the QD surface [42,43].

According to the FTIR spectra, the surface ligand of InP QDs changes from amine to OA after either ZnOA or CdOA treatment. Oleates may form a complex with the QD metal sites in different modes, for example, monodentate, chelating bidentate, and bridging bidentate [44,45]. The difference in stretching vibration may identify the complexation (∆(ν_as_-ν_s_)), as listed in Table S4 and Scheme S1. The above three binding modes exist in both ZnOA- and CdOA-InP QDs, indicating that oleates could bind to the QD metal sites in multiple configurations.

**Figure 5:**
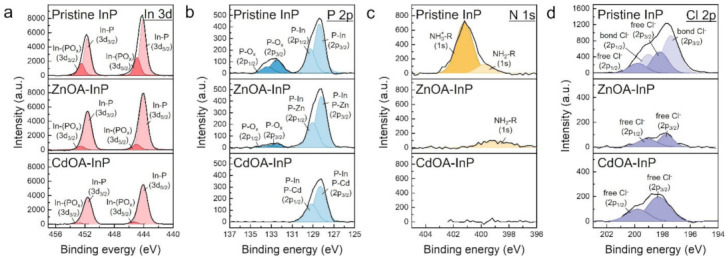
XPS (**a**) In 3d, (**b**) P 2p, (**c**) N 1s and (**d**) Cl 2p spectra of pristine, ZnOA-, and CdOA-InP QDs.

XPS In 3d spectra of pristine, ZnOA- and CdOA-InP QDs are shown in Figure 5a. The corresponding XPS survey spectra are exhibited in Figure S1. Pristine InP QDs show a doublet peak at 444.2 and 451.8 eV with a spin-orbit splitting of 7.6 eV between 3d_5/2_ and 3d_3/2_ (top spectrum). The peaks have two sub-doublets with one at 444.1 and 451.7 eV (light red doublet) assigned to In-P and another at 445.0 and 452.6 eV ascribed to In-(PO_x_) (dark red doublet) [46]. After metal oleate treatments, the ZnOA- (middle spectrum) and CdOA-InP QDs (bottom spectrum) have narrower In 3d peaks than those of the pristine ones, and both doublets are located at 444.0 and 451.6 eV. The narrowed In 3d peaks of ZnOA- and CdOA-InP QDs show a more prominent In-P sub-doublet and decreased In-(PO_x_) sub-doublet. Figure 5b shows P 2p spectra. Pristine InP QDs (top spectrum) have two separated peaks assigned to P atoms in different chemical states, as shown by the peaks at 128.4 eV (light blue doublet, P-In) and 132.6 eV (dark blue doublet, P-O_x_, oxidized InP, InPO_x_) [47,48]. Both peaks show a spin-orbit splitting of 0.87 eV between 2p_3/2_ and 2p_1/2_. The calculated InPO_x_/InP integrated area ratio from P 2p spectrum of pristine InP QDs is 0.3. After the metal oleate treatment, ZnOA- (middle spectrum) and CdOA-InP QDs (bottom spectrum) show decreased InPO_x_/InP ratio of 0.08 and nearly zero, respectively. The existence of InPO_x_ in pristine InP QDs is a result of inevitably native oxide induced by air exposure, trace water, or side-reactions in InP QD growth [26]. For ZnOA- and CdOA-InP QDs, the relative disappearance of InPO_x_ signals in both In 3d and P 2p spectra prove that metal oleate precursors remove the surface InPO_x_ layer. Oxidation of ZnOA-InP QDs occurs during sample transportation to the XPS measurement. According to Pearson’s hard-soft-acid-base (HSAB) theory [49], the soft base P forms a stronger binding with the soft acid Cd, and the soft P forms a weaker binding with borderline acid Zn [50]. Thus, the absence of oxidation of CdOA-InP QDs can be ascribed to the stronger P-Cd binding, impeding the oxidation. P-Zn and P-Cd bonds exist in P 2p spectra of ZnOA- and CdOA-InP QDs, respectively. However, the P-Zn and P-Cd bonds highly overlap with P-In bonds and thus become indistinguishable, as the fitting curves shown in Figure S5.

Furthermore, QD surface ligand exchange after metal oleate treatment is verified by N 1s and Cl 2p spectra, as displayed in Figure 5c,d. The pristine InP QDs feature an oleylammonium chloride ([OAmH^+^]-Cl^−^) ion pair surface ligand, as reported in our previous work [51,52]. The ZnOA- and CdOA-InP QDs show vastly decreased peak intensities in both N 1s and Cl 2p spectra, implying removal of [OAmH^+^]-Cl^−^ ligands. Besides, both ZnOA- and CdOA-InP QDs observe a rise of intensities for O 1s with Zn-O or Cd-O binding and O-C-O/O-C=O from OA ligand (Figure S6a) [53,54,55]. The O-C-O/O-C=O binding in OA can also be found in C 1s spectra (Figure S6b) [56,57]. Increased Zn content in ZnOA-InP QDs and Cd in CdOA-InP QDs are also noticed (Figure S6c and Figure S7). The results confirm that ZnOA- and CdOA-InP QDs are actually passivated by ZnOA and CdOA, respectively. Moreover, long alkyl OA chains of ZnOA and CdOA can superior passivate QD surfaces and reduce the environmental influence, leading to an observable oxide-free QD surface.

Surface oxidation on InP QDs can be further supported by ^31^P SSNMR analysis (Figure S8). The resonance peak evidences an increase or decrease of surface oxidation at ~ 5 ppm assigned to InPO_x_ and the resonance at ~−200 ppm ascribed to InP [58]. The results from ^31^P SSNMR spectra of all InP QDs are consistent with those derived from the XPS analysis. Furthermore, surface passivation of InP QDs by metal oleate is supported by increased PL lifetime of corresponding InP QDs in TRPL spectra (Figure S9 and Table S5).

**Figure 6:**
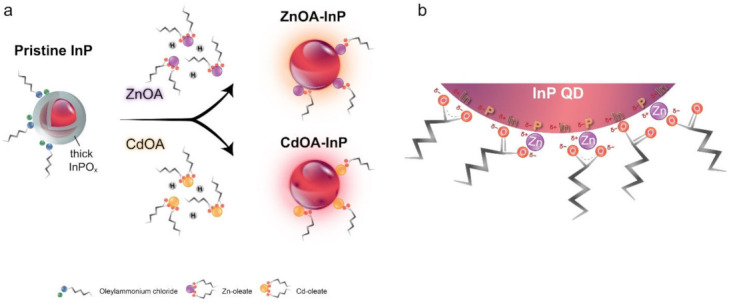
(**a**) A sketch illustrating the surface passivation and surface oxide etching effect of ZnOA and CdOA on pristine InP QDs. (**b**) Possible binding motifs of metal oleates on InP QD surface.

Effect of surface passivation and surface oxide etching from metal oleates is schematically illustrated in Figure 6a. It is known that metal oleate is a carboxylate complex, which is able to passivate nanocrystals and improve the optical properties [59]. Moreover, InP has a sensitive nature and would facilely be affected by other chemicals; therefore, according to the above analyses, the surface chemistry of InP QDs is described as follows. Pristine InP QDs with a surface InPO_x_ oxide layer is capped by [OAmH^+^]-Cl^−^ ion pair ligands; after metal oleate treatment, either ZnOA or CdOA with bifunctionality can remove the InPO_x_ layer and passivate the QD surface with metal ions. However, as stated above, an over-etch effect could exist in CdOA-InP QDs with some additional surface defects. Some possible binding motifs of metal oleates on the QD surface are illustrated in Figure 6b. A metal oleate may simply passivate the P atom or simultaneously passivate both P and In atoms. In addition, some excess OA in metal oleate precursors could also straightaway bind to the surface In atoms. Consequently, ZnOA- and CdOA-InP QDs could preserve the relative oxide-free surface because of the long alkyl metal oleates and well-passivated QD surface P and In atoms. The FTIR analysis also supports the complicated surface binding motifs of metal oleates.

### 3.4. Material Stability and ZnSe/ZnS Shelling of InP QDs

**Figure 7:**
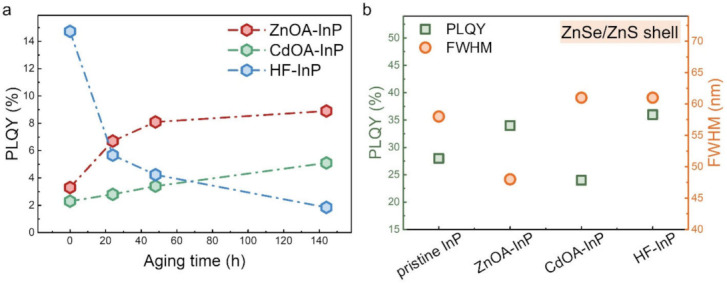
(**a**) Material stability of ZnOA−, CdOA−, and HF−InP QDs examined by PLQY evolution against an atmosphere aging process for 144 h. (**b**) PLQY and FWHM of shelled (ZnSe/ZnS shell) pristine, ZnOA-, CdOA-, and HF-InP QDs.

The material stability of InP QDs is examined by aging them in air atmosphere. The evolution of PLQY against aging time is given in Figure 7a. Corresponding PL and optical absorption spectra may be found in Figure S10. After 144 h of aging, ZnOA-InP QDs observe a nearly unchanged absorption curve and PLQY gradually increases up to 8.9%; the PLQY of CdOA-InP QDs extends to 5.1%, but HF-InP QDs see rapid decreases in PLQY from 14.7% to 1.8%. Increases in the PLQYs of ZnOA- and CdOA-InP QDs may be attributed to better surface passivation caused by surface ligand rearrangement [8,60]. Furthermore, the absorption curve broadens, alongwith a large blueshift (Figure S10e); and PLQY rapidly decreases in the case of HF-InP QDs, indicating that HF-InP QDs are rather unstable when subject to atmospheric conditions for a long time. Compared to HF-InP QDs, ZnOA and CdOA-InP QDs are more durable due to the passivation of their surface with long alkyl metal oleates that also prevent an environmental influence and maintain PLQYs.

HF-treated and metal oleate-treated InP QDs are further overcoated with an ZnSe/ZnS shell. The optical properties of shelled InP QDs are shown in Figure 7b. Corresponding PL spectra and a summary of optical properties are given in Figure S11 and Table S6. For pristine InP QDs, the PLQY increases from ~0.1% to 27.1% after growing with a ZnSe/ZnS shell, while the PL FWHM remains similar (~57 nm). The PLQY of shelled ZnOA-InP QDs increases to 34.4%, which is comparable with that of shelled HF-InP QDs (~35.7%). Notably, shelled ZnOA-InP QDs possess ~20% narrower PL FWHM (~48 nm) than either shelled pristine InP (~58 nm) or shelled HF-InP (~61 nm). This indicates that the ZnOA treatment does not affect the shell growth, suitable for preparing InP cores or core/shell QDs. Compared with the HF treatment, the ZnOA treatment provides much narrower FWHM and more stable InP cores. Note that the shell growth is not optimized and the PLQY may be further improved. Shelled CdOA-InP QDs have a lower PLQY (~24%) and broader FWHM (~61 nm), which is ascribed to over-etching of InP cores that produces extra surface defects, supported by a tail emission band relating to the defect states in their PL spectrum (Figure S11). The results show the potential of ZnOA treatment as an alternative method to remove InP surface oxide with higher safety and a feasible operation process. The PLQY of the QDs in this study was not optimized as the QDs in some processes might be exposed to air some time, e.g., centrifugation and some surface treatments. A future study may be focused on preparation in a moisture- and oxygen-free environment.

## 4. Conclusions

This paper demonstrates bifunctional metal oleate treatment for aminophosphine-based InP QDs to simultaneously passivate QD surface and remove the surface oxide layer. With the surface passivation effect, the PLQY of InP QDs increases from ~0.1% to as high as ~9% through a ZnOA treatment and an atmosphere aging process without growing any type-I shell. The etching effect is evidenced by XPS analysis with decreased InPO_x_ oxide signals. Compared to a rapid decay of the HF passivation, the long alkyl metal oleates provide a more stable surface passivation effect on InP QDs, maintaining the oxide-free surface of QDs. Moreover, removal of the defective surface oxide layer on ZnOA-InP cores leads to a ~20% narrower PL FWHM of ZnOA-InP/ZnSe/ZnS QDs. This protocol demonstrates the potential of bifunctional metal oleate treatment as a safer and alternative method for HF treatment, which can be used to remove the surface oxide and improve the optical properties for aminophosphine-based InP QDs.

## Data Availability

Not applicable.

## Supplementary Materials

The following supporting information can be downloaded at: https://www.mdpi.com/article/10.3390/nano12030573/s1. Figure S1: XPS survey spectra of pristine InP, ZnOA-InP, CdOA-InP and HF-InP QDs. Figure S2: (a) Optical absorption spectra of pristine InP QDs in an OA/toluene solution with the OA/InP molar ratio of 0.5, lasting for 5 min. The pristine InP QDs are in a concentration of 10 mM in toluene. The broadened and blueshifted absorption curve of the sample suggests that OA has some etching effect on pristine InP QDs. If the pristine InP QDs are mixed with OA for more than 10 min, the QD solution becomes a transparent solution without detectable absorption peak. (b) Control samples of InP QDs after a post-treatment with only ZnCl_2_ or Zn(ac)_2_ as metal salts and without OA are prepared. The InP QDs are first purified with acetone to remove reaction residuals. Then, ZnCl_2_ and are dissolved in ODE, respectively, then added into 10 mM InP QD solution in a Zn/InP QD concentration of 20. The results show that the only addition of metal salts without OA lead to nearly no change in InP QD optical properties. Figure S3: PL spectra of (a) ZnOA- and (b) CdOA-InP QDs with various metal oleate to InP ratio. Figure S4: XRD patterns of pristine, ZnOA- and CdOA- InP QDs in which all diffraction peaks are corresponded to InP cubic structure. The weak diffraction intensities of CdOA-InP QDs indicate a lower crystallinity of CdOA-InP QDs. Figure S5: XPS P 2p spectra of (a) ZnOA- and (b) CdOA-InP QDs involving P-In, P-Zn and P-Cd doublets in the spectra. Though the P-Zn and P-Cd doublets would exist in the spectra, the P-In doublet highly overlap with P-Zn and P-Cd doublets, making them distinguishable. Figure S6: XPS (a) O1s, (b) C 1s and (c) Zn 2p_3/2_ spectra of pristine, ZnOA- and CdOA-InP QDs. Figure S7: XPS Cd 3d spectra of CdOA-InP QDs indicating surface passivation by CdOA. Figure S8:**^31^**P MAS SSNMR spectra of pristine, ZnOA-, CdOA- and HF-InP QDs. Figure S9: Time-resolved PL (TRPL) spectra of pristine, ZnOA-, CdOA- and HF-InP QDs. Figure S10: Evolution of (a), (c), (e) optical absorption and (b), (d), (f) PL spectra of ZnOA-, CdOA- and HF-InP QDs in atmosphere aging process for 144 h. Figure S11: PL spectra of ZnSe/ZnS shelled pristine, ZnOA-, CdOA- and HF-InP QDs. Table S1: Gibbs free energy of reactions including phosphines oxidized to phosphorous oxides. Table S2: Standard enthalpy of formation and standard molar entropy used to calculate Gibbs free energy of reaction. Table S3: Standard Gibbs free energy of formation of the various compounds. Table S4: Symmetric (ν_s_(COO^−^)) and asymmetric (ν_as_(COO^−^)) carboxylate stretches, ∆ν and proposed metal oleate binding modes of ZnOA- and CdOA-InP QDs determined by FTIR. Table S5: Fitting parameters for TRPL decay dynamics in pristine, ZnOA-, CdOA- and HF-InP QD samples. Table S6: Summary of PL peak, FWHM and PLQY of shelled pristine, ZnOA-, CdOA- and HF-InP QDs. Scheme S1: Illustration of three different carboxylate binding modes with metal ions (M) including chelating bidentate, bridging bidentate and monodentate. References [61,62,63,64,65,66,67,68,69,70,71,72] are cited in the Supplementary Materials.
