# Supplementary material for: Bifunctional Metal Oleate as an Alternative Method to Remove Surface Oxide and Passivate Surface Defects of Aminophosphine-Based InP Quantum Dots
Source: Nanomaterials (Basel). 2022 Feb 8;12(3):573. doi: 10.3390/nano12030573 (PMC8838112; doi:10.3390/nano12030573)
Supplement: Supplementary file 1 [file nanomaterials-12-00573-s001.zip › nanomaterials-1579400-supplementary.pdf]

*nanomaterials*

*Supplementary Materials*

# Bifunctional Metal Oleate as an Alternative Method to Remove Surface Oxide and Passivate Surface Defects of Aminophosphine-based InP Quantum Dots

**Pin-Ru Chen, Minh-Son Hoang, Kuo-Yang Lai and Hsueh-Shih Chen ***

Department of Materials Science and Engineering, National Tsing Hua University, Hsinchu 30013, Taiwan; sophia801004@gmail.com (P.-R.C.); hmson.88@gmail.com (M.-S.H.); harry9703011345@gmail.com (K.-Y.L.)
* Correspondence: chenhs@mx.nthu.edu.tw

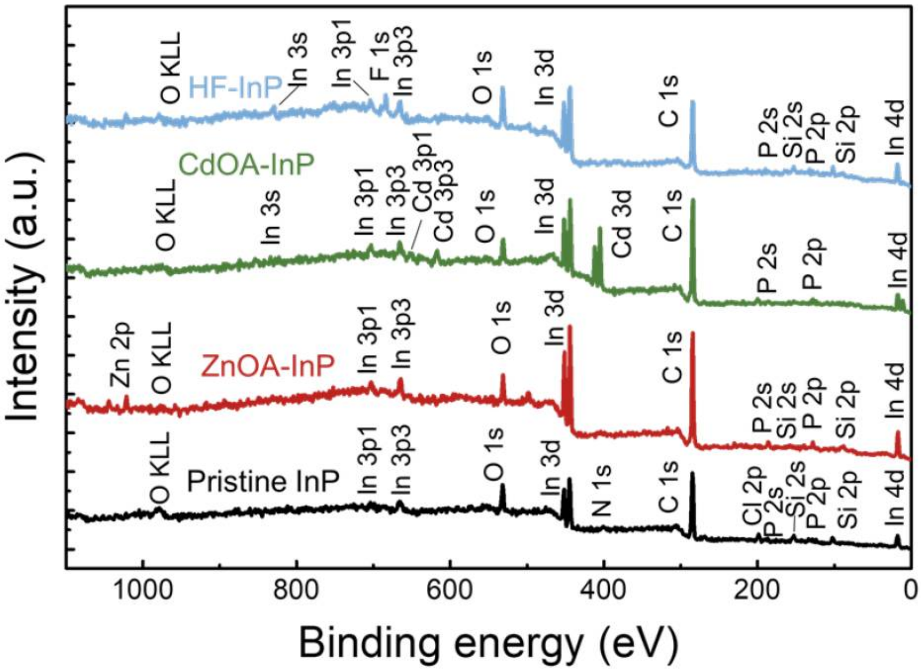


**Figure S1.** XPS survey spectra of pristine InP, ZnOA-InP, CdOA-InP and HF-InP QDs.

**Table S1.** Gibbs free energy of reactions including phosphines oxidized to phosphorous oxides.

| Reaction | $\Delta_{rxn}G°_{298}$ (kJ/mol) |
|---|---|
| $4PH_3(g)+8O_2(g)\rightarrow P_4O_{10}(s)+6H_2O(l)$ | −4095 |
| $4PH_3(g)+8O_2(g)\rightarrow P_4O_{10}(s)+6H_2O(g)$ | −4043 |
| $2P_2H_4(l)+7O_2(g)\rightarrow P_4O_{10}(s)+4H_2O(l)$ | −3729 |
| $2P_2H_4(l)+7O_2(g)\rightarrow P_4O_{10}(s)+4H_2O(g)$ | −3708 |
| $2P_4H_2(s)+11O_2(g)\rightarrow 2P_4O_{10}(s)+2H_2O(l)$ | −5843 |
| $2P_4H_2(s)+11O_2(g)\rightarrow 2P_4O_{10}(s)+2H_2O(g)$ | −6025 |
| $4PH_3(g)+6O_2(g)\rightarrow P_4O_6(s)+6H_2O(l)$ | −2941 |
| $4PH_3(g)+6O_2(g)\rightarrow P_4O_6(s)+6H_2O(g)$ | −2890 |
| $2P_2H_4(l)+5O_2(g)\rightarrow P_4O_6(s)+4H_2O(l)$ | −2589 |
| $2P_2H_4(l)+5O_2(g)\rightarrow P_4O_6(s)+4H_2O(g)$ | −2544 |
| $2P_4H_2(s)+7O_2(g)\rightarrow 2P_4O_6(s)+2H_2O(l)$ | −3536 |
| $2P_4H_2(s)+7O_2(g)\rightarrow 2P_4O_6(s)+2H_2O(g)$ | −3520 |

**Table S2.** Standard enthalpy of formation and standard molar entropy used to calculate Gibbs free energy of reaction.

| Compound | $\Delta_fH°$(J/mol) | S°(J/mol·K) | Ref. |
|---|---|---|---|
| $PH_3(g)$ | 5430 | 209.9 | [61] |
| $P_2H_4(l)$ | 35,300 | 167.4 | [61,62] |
| $P_4H_2(s)$ | 28,840 | 167.2 | [61] |
| $H_2O$ (l) | −285,830 | 69.9 | [63] |
| $H_2O$ (g) | −241,830 | 188.8 | [63] |
| $O_2$ (g) | 0 | 205.2 | [64] |
| $P_4O_6$ (s) | −1,593,700 | 346.9 | [65] |
| $P_4O_{10}$ (s) | −2,904,090 | 228.9 | [66] |

**Table S3.** Standard Gibbs free energy of formation of the various compounds.

| Bond | Compound | $\Delta_fG°_{298}$ (J/mol) | Ref. |
|---|---|---|---|
| P-H | $PH_3(g)$ | 13,500 | [67] |
| P-O | $P_4O_{10}$ (s) | −2,687,000 | [68] |
| P-Zn | $Zn_3P_2$ (s) | −158,707 | [69] |
| In-F | $InF_3$ (s) | −114,790 | [70] |
| In-O | $In_2O_3$ (s) | −830,800 | [67] |

The standard enthalpy of formation used to calculate standard Gibbs free energy of formation could be derived from the bond energy required to break and produce. Thus, the standard Gibbs free energy of formation can be an indirect evidence for the stability of a specific bond.

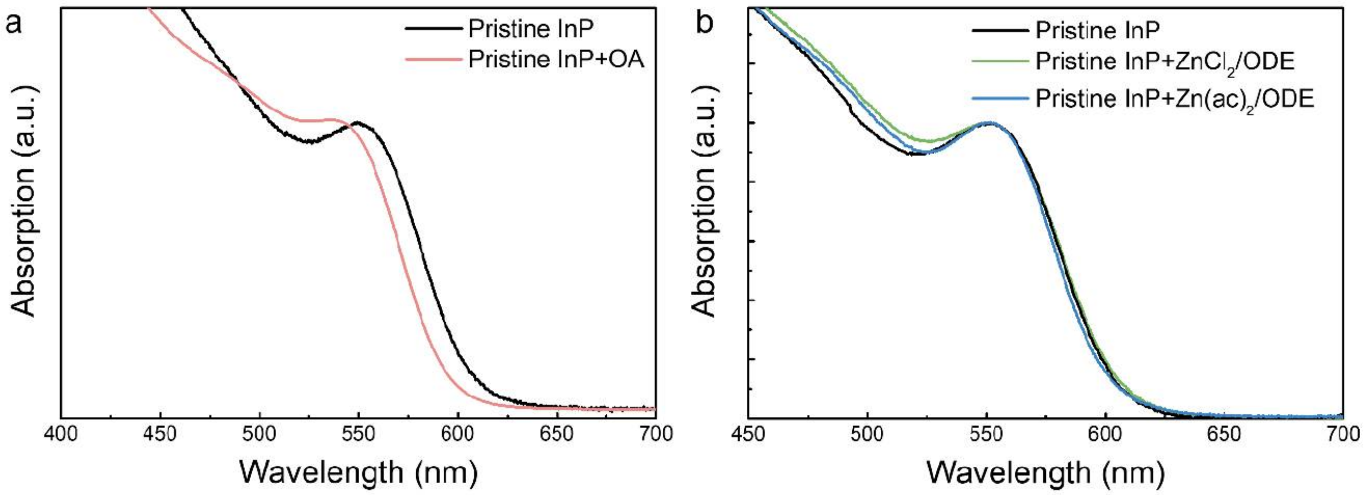


**Figure S2.** (a) Optical absorption spectra of pristine InP QDs in an OA/toluene solution with the OA/InP molar ratio of 0.5, lasting for 5 min. The pristine InP QDs are in a concentration of 10 mM in toluene. The broadened and blueshifted absorption curve of the sample suggests that OA has some etching effect on pristine InP QDs. If the pristine InP QDs are mixed with OA for more than 10 min, the QD solution becomes a transparent solution without detectable absorption peak. (b) Control samples of InP QDs after a post-treatment with only $ZnCl_2$ or $Zn(ac)_2$ as metal salts and without OA are prepared. The InP QDs are first purified with acetone to remove reaction residuals. Then, $ZnCl_2$ and are dissolved in ODE, respectively, then added into 10 mM InP QD solution in a Zn/InP QD concentration of 20. The results show that the only addition of metal salts without OA lead to nearly no change in InP QD optical properties.

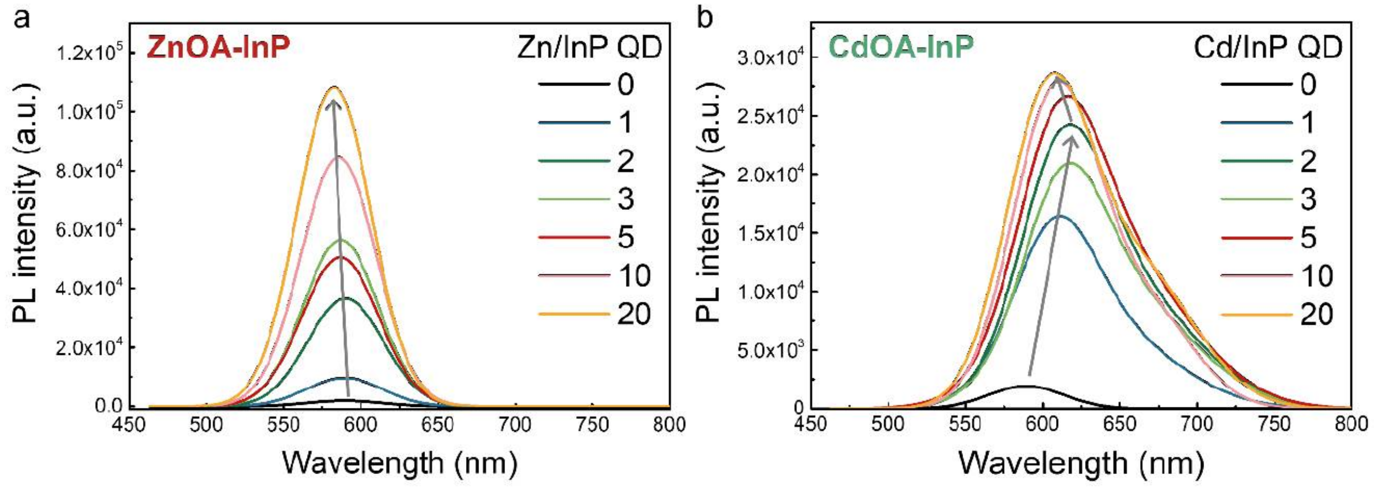


**Figure S3.** PL spectra of (a) ZnOA- and (b) CdOA-InP QDs with various metal oleate to InP ratio.

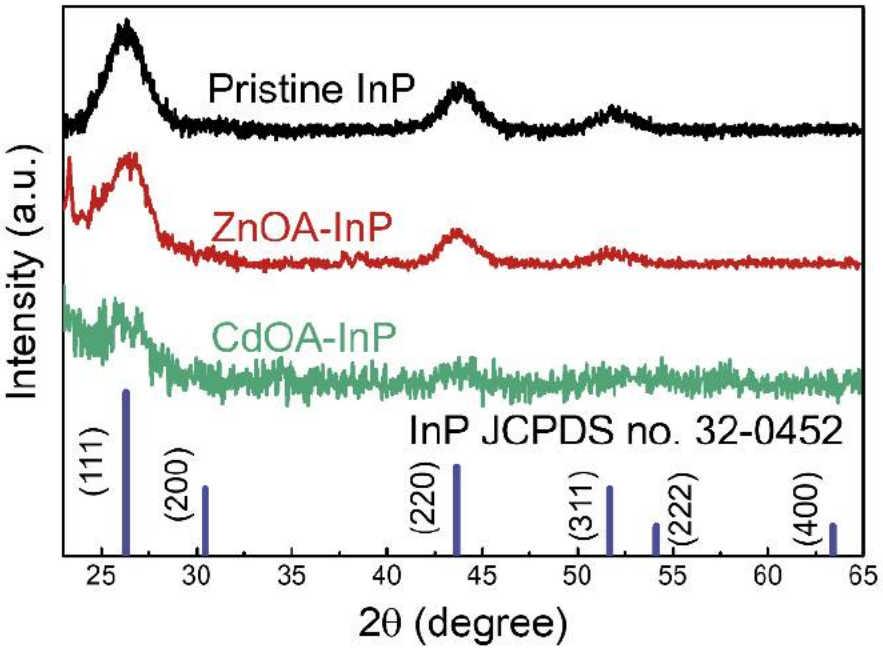


**Figure S4.** XRD patterns of pristine, ZnOA- and CdOA- InP QDs in which all diffraction peaks are corresponded to InP cubic structure. The weak diffraction intensities of CdOA-InP QDs indicate a lower crystallinity of CdOA-InP QDs.

**Table S4.** Symmetric ($\nu_s(COO^-)$) and asymmetric ($\nu_{as}(COO^-)$) carboxylate stretches, $\Delta\nu$ and proposed metal oleate binding modes of ZnOA- and CdOA-InP QDs determined by FTIR.

| | $\nu_s(COO^-)$ (cm$^{-1}$) | $\nu_{as}(COO^-)$ (cm$^{-1}$) | $\Delta\nu$ (cm$^{-1}$) | Binding Mode |
|---|---|---|---|---|
| ZnOA-InP | 1408 | 1525 | 117 | chelating bidentate |
| | | 1548 | 140 | bridging bidentate |
| | | 1621 | 213 | monodentate |
| CdOA-InP | 1410 | 1532 | 122 | chelating bidentate |
| | | 1550 | 140 | bridging bidentate |
| | | 1641 | 231 | monodentate |

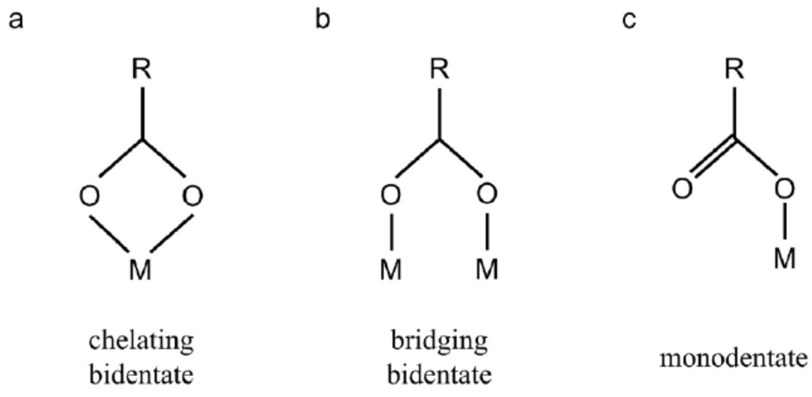


**Scheme 1.** Illustration of three different carboxylate binding modes with metal ions (M) including chelating bidentate, bridging bidentate and monodentate.

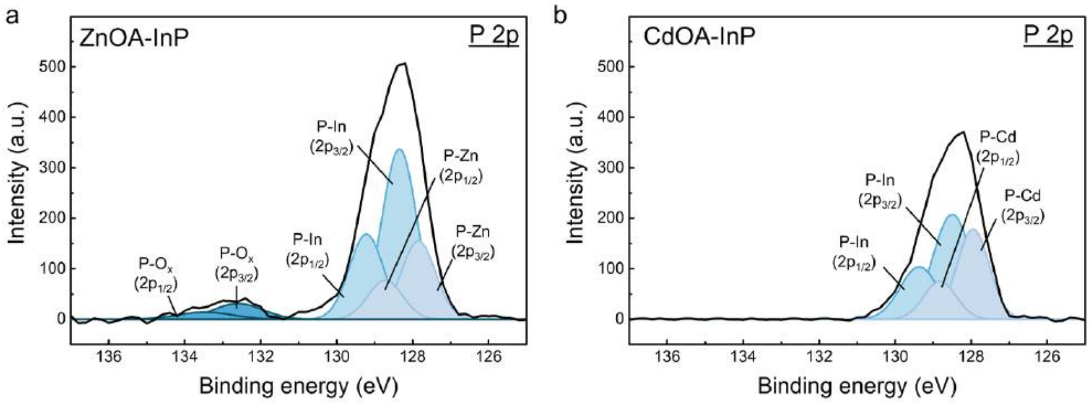


**Figure S5.** XPS P 2p spectra of (a) ZnOA- and (b) CdOA-InP QDs involving P-In, P-Zn and P-Cd doublets in the spectra. Though the P-Zn and P-Cd doublets would exist in the spectra, the P-In doublet highly overlap with P-Zn and P-Cd doublets, making them distinguishable.

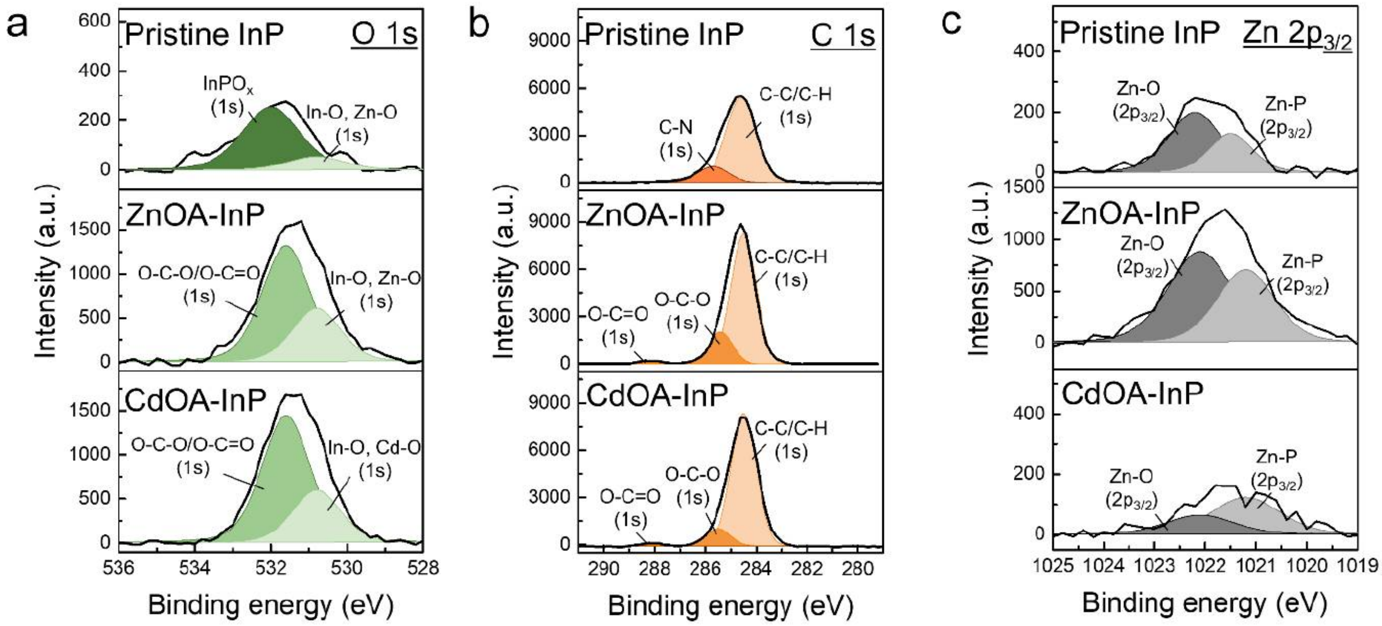


**Figure S6.** XPS (a) O1s, (b) C 1s and (c) Zn 2p3/2 spectra of pristine, ZnOA- and CdOA-InP QDs.

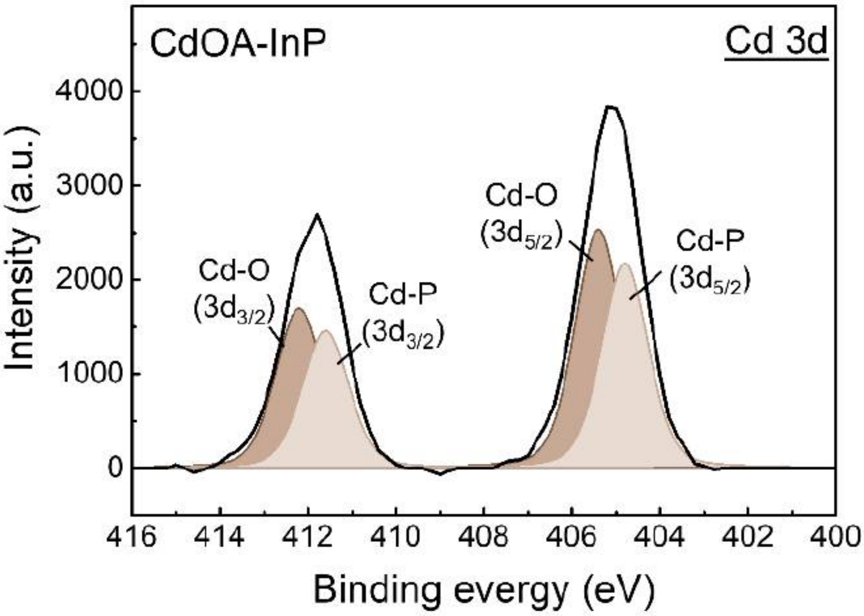


**Figure S7.** XPS Cd 3d spectra of CdOA-InP QDs indicating surface passivation by CdOA.

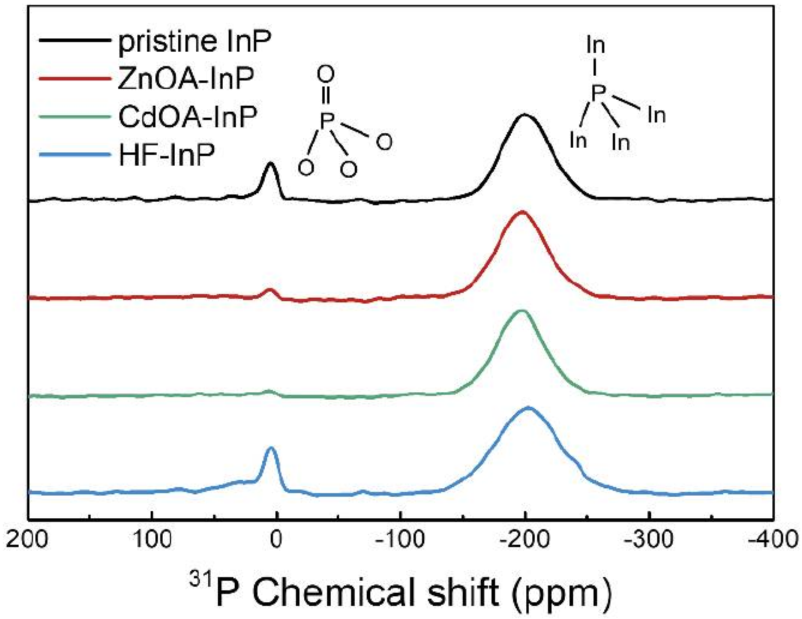


**Figure S8.** $^{31}P$ MAS SSNMR spectra of pristine, ZnOA-, CdOA- and HF-InP QDs. The single resonance at −200 ppm is ascribed to InP while the resonance at ~ 5 ppm is assigned to oxidized InP ($InPO_x$) [58]. The results show extremely low $InPO_x$ of ZnOA- and CdOA-InP QDs suggesting effectively removal of InPOx and preserve of the oxide-free surface by metal oleate treatment. An increased oxidation is observed in HF-InP QD sample indicating re-oxidation of QD surface after the HF treatment.

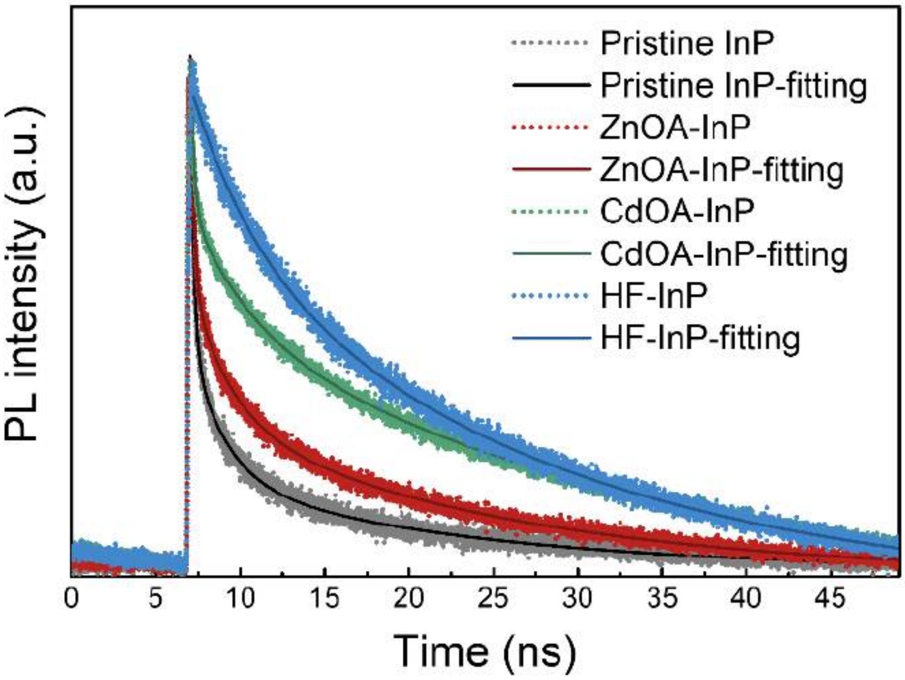


**Figure S9.** Time-resolved PL (TRPL) spectra of pristine, ZnOA-, CdOA- and HF-InP QDs. The PL decay curves can be fitted with a bi-exponential function: $I(t)=A_1\exp(\frac{-t}{\tau_1})-+A_2\exp(\frac{-t}{\tau_2})$, where I is the PL intensity at time t, $A_1$ and $A_2$ are weight constants, $A_1+A_2=1$, and $\tau_1$ and $\tau_2$ are time constants for the exponential components. The weighted PL lifetime ($\tau_{av}$) can be determined by the expression: $\tau_{av}=(A_1\tau_1^2+A_2\tau_2^2)/(A_1\tau_1+A_2\tau_2)$. The short lifetime component ($\tau_1$) is ascribed to band-edge emission while the long lifetime component ($\tau_2$) is assigned to surface defect states [71,72]. The PL lifetime of pristine, ZnOA-, CdOA- and HF-InP are 5.77, 10.37, 17.87 and 19.91 ns, respectively. The increased PL lifetime of ZnOA-, CdOA- and HF-InP QDs are attributed to the surface passivation through either metal oleate and HF treatment. Compared with ZnOA-InP QDs, the longer $\tau_{av}$ lifetime of CdOA-InP QDs is related to the introduced surface defect states after CdOA treatment.

**Table S5.** Fitting parameters for TRPL decay dynamics in pristine, ZnOA-, CdOA- and HF-InP QD samples.

| Sample | $A_1$ | $\tau_1$ (ns) | $A_2$ | $\tau_2$ (ns) | Weighted $\tau_{av}$ (ns) |
|---|---|---|---|---|---|
| Pristine InP | 0.91 | 0.39 | 0.09 | 8.32 | 5.77 |
| ZnOA-InP | 0.72 | 0.53 | 0.28 | 11.55 | 10.37 |
| CdOA-InP | 0.69 | 0.92 | 0.31 | 19.62 | 17.87 |
| HF-InP | 0.27 | 3.74 | 0.73 | 20.99 | 19.91 |

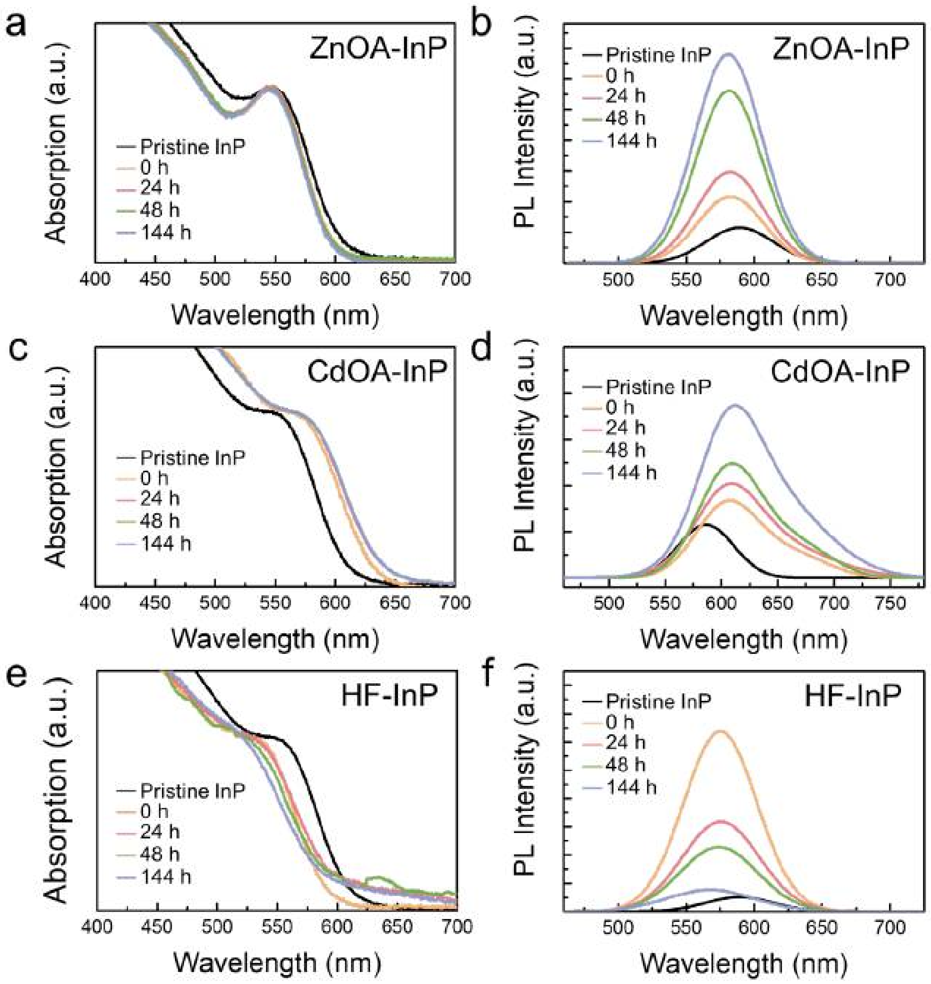


**Figure S10.** Evolution of (a), (c), (e) optical absorption and (b), (d), (f) PL spectra of ZnOA-, CdOA- and HF-InP QDs in atmosphere aging process for 144 h.

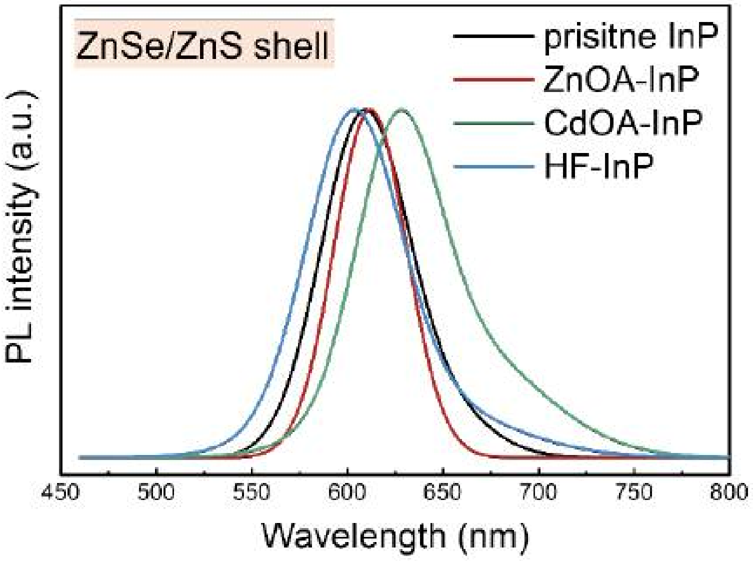


**Figure S11.** PL spectra of ZnSe/ZnS shelled pristine, ZnOA-, CdOA- and HF-InP QDs.

**Table S6.** Summary of PL peak, FWHM and PLQY of shelled pristine, ZnOA-, CdOA- and HF-InP QDs.

| **Sample** | **PL peak (nm)** | **FWHM (nm)** | **PLQY (%)** |
| --- | --- | --- | --- |
| Pristine InP/ZnSe/ZnS | 610 | 58 | 27.1 |
| ZnOA-InP/ZnSe/ZnS | 612 | 48 | 34.4 |
| CdOA-InP/ZnSe/ZnS | 628 | 61 | 24.3 |
| HF-InP/ZnSe/ZnS | 603 | 61 | 35.7 |
